# An epidemiological analysis of acute flaccid paralysis in Khuzestan Province, southwest Iran, from 2006 to 2010

**DOI:** 10.4178/epih.e2016030

**Published:** 2016-07-19

**Authors:** Ali Akbar Momen, Abdolhussein Shakurnia

**Affiliations:** 1 Department of Pediatric, Faculty of Medicine, Ahvaz Jundishapur University of Medical Sciences, Ahvaz, Iran; 2 Department of Immunology, Faculty of Medicine, Ahvaz Jundishapur University of Medical Sciences, Ahvaz, Iran

**Keywords:** Epidemiology, Acute flaccid paralysis, Poliomyelitis, Iran, Khuzestan

## Abstract

**OBJECTIVES:**

Investigations into the epidemiology of acute flaccid paralysis (AFP) are an essential strategic component of the Global Poliomyelitis Eradication Initiative of the World Health Organization (WHO), and are part of the certification process for polio eradication worldwide. This is an epidemiological report of AFP incidence in children less than 15 years old in southwest Iran.

**METHODS:**

This was a retrospective cohort study, carried out based on WHO guidelines, in which we reviewed non-polio AFP cases recorded from January 2006 to December 2010 in different regions of Khuzestan Province, southwest Iran. In this study, the records of all children under 15 years old with AFP were evaluated.

**RESULTS:**

During a 5-year period, 137 cases of AFP were reported (incidence rate, 2.21 per 100,000 children <15 years old). More than 50% (73 of 137) of the cases were boys, and 52.6% (72 of 137) were under 5 years of age, with a mean age of 5.39±3.98 years. The incidence of AFP was significantly higher in older children (p=0.001). The most common cause of paralysis was Guillain-Barré syndrome (117 of 137). None of the cases were diagnosed with acute poliomyelitis.

**CONCLUSIONS:**

In this study, we found that the incidence rate of AFP in the region was almost in agreement with the expected incidence of AFP in children less than 15 years old; therefore, the AFP surveillance program in Khuzestan Province is satisfactory in terms of reliability and effectiveness. Nevertheless, routine vaccination against polio and ensuring that patients with AFP receive follow-up are essential for eradicating polio.

## INTRODUCTION

Poliomyelitis is a highly contagious viral disease caused by poliovirus. In 1988, the World Health Organization (WHO) implemented the Global Polio Eradication Initiative (GPEI) with the goal of eradicating polio worldwide. In addition to attaining a high routine immunization coverage rate, surveillance of acute flaccid paralysis (AFP) is a key strategic component of the GPEI. In accordance with the goals of the WHO, a robust surveillance system for AFP reporting was established in Iran in 1991 [1,2].

AFP is a clinical syndrome that can be caused by a wide range of etiologies. AFP presents with a rapid onset of weakness, including weakness in the muscles involved in respiration and swallowing, and progresses to maximum severity within several days to weeks. Since AFP surveillance is a basic strategic component of polio eradication campaigns, it is an important responsibility of the public health system in most countries. AFP surveillance programs can be helpful in identifying high-risk areas or groups and in monitoring the status of polio in each geographical region [3,4].

Many diseases are considered relevant for the differential diagnosis of AFP. The most important conditions to consider are non-polio enteroviruses, Guillain-Barré syndrome (GBS), transverse myelitis, acute motor axonal neuropathy, and acute traumatic neuritis [5]. GBS has become the most common cause of AFP in all ages, especially in children. The annual global incidence rate of GBS is one to four cases per 100,000 people. GBS is a post-infectious polyneuropathy that mainly affects the motor nerve branches [6].

AFP is diagnosed clinically through a medical history and physical examination. The AFP surveillance program requires physicians to report all cases of AFP in children less than 15 years old to regional health centers, using AFP surveillance guidelines and standard forms. In these forms, the demographic data and the clinical and epidemiologic findings of the patient are recorded accurately and sent to the National Center for Polio Eradication [7].

The incidence rate of non-polio AFP in Iran and worldwide has been estimated in many studies. In Iran, Gharibzadeh et al. [8] reported non-polio AFP incidence rates ranging from 0.3 to 6.5 per 100,000, with an overall national rate of 2.9 per 100,000 children less than 15 years old. The global incidence rate of AFP varies from 0.8 per 100,000 children less than 15 years old in Australia to 4.0 per 100,000 children less than 15 years old in Afghanistan [9,10].

Since diagnosing and identifying cases of AFP in every country is a part of the global surveillance system for the eradication of poliomyelitis, studying AFP incidence in specific regions within individual countries is important. The current study was carried out to investigate the incidence of AFP and the effectiveness of the AFP surveillance program in Khuzestan Province, southwest Iran, from January 2006 to December 2010.

## MATERIALS AND METHODS

This retrospective cohort study was conducted on children less than 15 years old in Khuzestan Province. Khuzestan Province is located in southwest Iran, and has a hot and dry climate. The population of the province exceeds 4,500,000 people, including approximately 1,200,000 people less than 15 years old.

The surveillance database of AFP in children less than 15 years old based on reports to the central health office of Khuzestan Province from January 2006 to December 2010 was obtained and reviewed for this investigation. This study was approved by the ethical committee of the Ahvaz Jundishapur University of Medical Sciences.

The cases reviewed in this study included the records of all children less than 15 years old whose diagnosis of AFP had been reported to the provincial central health office. In Iran, a nationwide health system covering all regions of the country actively monitors the incidence of non-polio AFP. It is mandatory for healthcare workers to report and track all children with AFP. In the health centers where the cases are reported, members of a national expert committee evaluate all AFP cases according to standard methods recommended by the WHO [11]. Case investigation is done using a standard WHO form. Data are gathered using a questionnaire that includes the personal characteristics of the patient such as age, gender, place of residence, and vaccination status; as well as the clinical presentation of the disease, including signs and symptoms, primary impression, final diagnosis, time of the appearance of paralysis, presence of fever at the beginning of paralysis, symmetry of paralysis, the condition of the patient at a 60-day follow-up visit, the patient’s final outcome, and stool test results. According to the WHO guidelines for AFP surveillance systems, the definitive diagnosis of non-polio cases and poliomyelitis is made based on two stool cultures from children with AFP, with non-polio cases diagnosed based on negative cultures [7].

The yearly incidence rate of AFP was used to estimate the overall incidence rate during the study period. The yearly incidence rate was calculated by dividing the number of the AFP cases recorded each year by the total population under 15 years old in that year, which was obtained from the Statistical Center of Iran [12].The detected cases were divided into three different age groups including <5, 5-9, and 10-14 years old. In order to compare the frequency of AFP in different age groups and gender we used the chi-square (relative risk). The analyses were performed using SPSS version 18.0 (SPSS Inc., Chicago, IL, USA). The p-values below 0.05 were considered statistically significant. And p-values <0.05 were considered to indicate statistical significance in comparisons of subgroups defined by characteristics such as gender and age.

## RESULTS

A total of 137 cases of AFP were diagnosed in children less than 15 years old during the 5-year period of the study. Of these cases, 53.3% (73 of 137) were boys and 46.7% (64 of 137) were girls. The average age of the cases was 5.39±3.98 years old. More than half of them (52.6%) were less than 5 years old. The number of AFP patients increased from 2006 to 2010; during the 5 years of the study, 19, 18, 28, 34, and 38 cases of AFP were reported, respectively.

**Figure 1.**
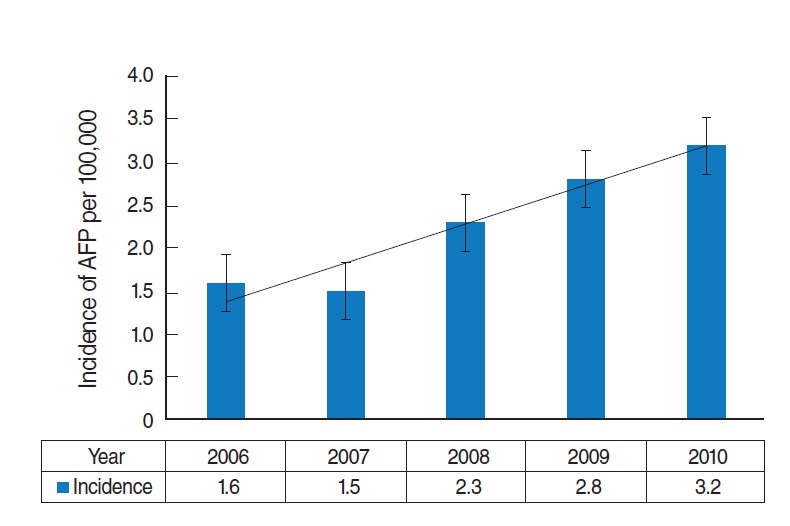
Incidence rate of non-polio acute flaccid paralysis (AFP) per 100,000 children under 15 years old in Khuzestan, Iran (2006-2010).

Based on reports from the Statistical Center of Iran [12], the population of children less than 15 years old in Khuzestan Province in the years of the study was 1,221,257; 1,226,167; 1,234,262; 1,245,627 and 1,260,363, respectively. Accordingly, the incidence rate in each year was calculated as the total number of cases per population of children less than 15 years old in that year, multiplied by 100,000 (Figure 1). The average AFP incidence rate in this 5-year period was calculated to be 2.21 per 100,000 children under 15 years old.

In Table 1, the incidence rate of AFP is shown according to gender, age, location, and season. No cases of AFP due to poliomyelitis were reported in this period. The most important causes of AFP were GBS (85.4%) and myositis (2.6%), with other causes accounting for the remaining 12% of cases, including tumors, brucellosis-associated septic arthritis, birth hypoxic-ischemic encephalopathy, transverse myelitis, and acute disseminated encephalomyelitis.

Comparisons of the risk of AFP incidence according to age and gender are presented in Table 2. According to Poisson regression analysis, the relative risk of AFP among children less than five years old was found to be almost two times greater than that observed in children five to nine years old and more than two times greater than that observed in children 10-14 years of age (p=0.002 and p=0.001, respectively). However, the AFP incidence rate did not differ significantly according to gender (p=0.34).

## DISCUSSION

The results of this study found the incidence rate of non-polio AFP in Khuzestan Province to be 2.21 per 100,000 children under 15 years old, which exceeds the WHO-established minimum AFP incidence rate. In addition, the increase in detected AFP cases from 2006 to 2010 suggests that surveillance indicators have improved within the 5-year period of the study.

Investigating and estimating the number of cases of non-polio AFP in different regional populations is the most important aspect for AFP surveillance and poliomyelitis-free certification worldwide. The WHO has established criteria for evaluating the performance of AFP surveillance, which include a non-polio AFP rate of at least one per 100,000 children less than 15 years of age [11]. Accordingly, the AFP incidence rate of 2.21 in 100,000 children under 15 years old indicates acceptable performance of the APF surveillance system in Khuzestan Province. Based on WHO reports in 2011, the estimated AFP incidence rate in Iran is 3.1%, which shows that the AFP surveillance system that has been implemented in Iran is appropriately sensitive, and suggests that this system reflects a fundamental accomplishment in the achievement and maintenance of polio eradication in Iran [13].

The findings of this study demonstrate that the detection of AFP cases increased from 2006 to 2010. It is worth emphasizing that this is a positive finding, as this increase is indicative of the sensitivity of the surveillance system in this region. Based on studies conducted in different parts of the country, the AFP incidence rate in Iran has risen from 2000 to 2012, which demonstrates that the surveillance system has improved [14,15].

In 2010, Gharibzadeh et al. [8] published a study on mapping the incidence of AFP in Iran and reported that the incidence rate of AFP ranged from 0.32 to 6.45 per 100,000. The functionality of the surveillance systems in each region is an influential factor contributing to the accurate identification of AFP cases. Therefore, accurate AFP reporting indicates the success of the surveillance and monitoring system against poliovirus.

In this study, GBS was the most common cause of AFP (85.4% of cases). A study carried out by Naeini et al. [4] in central Iran also reported GBS (83.5% of cases) to be the most common cause of AFP. Based on the findings of studies from Australia and Iraq, the frequency of GBS as the underlying cause of AFP is more than 50%, even in such disparate countries [1,16]. These findings are compatible with the results of our study. Since poliomyelitis has been controlled, GBS has become the most common cause of AFP worldwide.

The frequency of AFP was higher among boys than in girls (53.3% vs. 46.7%, respectively); but this difference was not statistically significant (p=0.34) Studies in Italy and Ghana have reported similar findings [17,18]. This indicates that gender does not significantly affect the incidence rate of AFP.

The results of the present study also showed that AFP incidence was significantly greater in younger children; in particular, it was approximately two times greater in children less than 5-year old than in other age groups. These findings are consistent with the results of similar studies. Studies in Turkey and Iran have likewise found the incidence of AFP to be significantly higher in children less than five years of age [19,20].

AFP surveillance has become known as the most reliable method for identifying possible poliomyelitis cases and verifying the eradication of poliovirus [5]. This method requires physicians and health care team members to perceive its importance, and to report potential cases as soon as possible.

By optimizing the coverage of polio vaccinations and strictly implementing a vaccination program in Iran, no cases of poliomyelitis have been reported in Iran since 2001 [1]. However, as poliomyelitis is endemic in Pakistan and Afghanistan (two neighboring countries on the eastern border of Iran), and given the presence of abundant travel and immigration from these countries to Iran, the spread of the disease is possible; therefore, strict implementation of this surveillance system is strongly recommended, especially in provinces and cities located close to the relevant borders.

This study has certain limitations. Standard WHO diagnostic criteria were applied retrospectively based on field reports by members of the Iranian health care system, and the children could not be evaluated or examined by the researchers. Regarding to diagnosis based on field reports by Iranian health centers, the data validity of the surveillance system would have somewhat influence on the findings of study. That’s why, like other similar studies that used AFP surveillance system data, the validity of the surveillance system would have influence on the results.

The results of this study showed that the AFP surveillance program in Khuzestan Province was efficient and delivered desirable results over the 5-year study period. The AFP incidence rate in children under 15 years old in the Province was greater than the rate expected by the WHO. Nevertheless, efforts should be made not only to maintain the existing process, but also to make sure that all cases of AFP are reported and investigated as soon as possible. Additionally, estimating the AFP incidence rate periodically in different regions is recommended in order to monitor the surveillance system and to ensure the continued eradication of poliovirus in Iran.

## Figures and Tables

**Table 1.** Frequency of non-polio acute flaccid paralysis by year, gender, age, location of residence, season of incidence, and clinical condition in Khuzestan, Iran, 2006-2010

Variable	Year					Total
	2006	2007	2008	2009	2010	
Gender						
Men	4(21.2)	6 (33.3)	18 (64.3)	24 (70.5)	21 (55.3)	73 (53.3)
Women	15 (78.8)	12 (66.6)	10 (35.7)	10 (29.4)	17 (44.7)	64 (46.7)
Age (yr)						
0-4	12 (63.2)	7 (38.9)	19 (67.9)	17 (50)	17 (44.7)	72 (52.6)
5-9	4(21.1)	7 (38.9)	8 (28.6)	9 (26.5)	10 (26.3)	38 (27.7)
10-14	3 (15.8)	4 (22.2)	1 (3.6)	8 (23.5)	11 (28.9)	27 (19.7)
Location of residence						
Urban	12 (63.2)	12 (66.7)	5(41.7)	15 (62.5)	27 (71.1)	71 (64.0)
Rural	7 (36.8)	6 (33.3)	7 (58.3)	9 (37.5)	11 (28.9)	40 (36.0)
Season of incidence						
Spring	3 (15.8)	4 (22.2)	7 (25.0)	11 (32.4)	10 (26.3)	35 (25.5)
Summer	1 (5.3)	4 (22.2)	6(21.4)	5 (14.7)	5 (13.2)	21 (15.3)
Fall	8 (42.1)	7 (38.9)	9 (32.1)	9 (26.5)	14 (36.8)	47 (34.4)
Winter	7 (36.8)	3 (16.7)	6(21.4)	9 (26.5)	9 (23.7)	34 (24.8)
Clinical condition						
Fever	9 (47.4)	12 (66.7)	15 (53.6)	16 (47.1)	17 (44.7)	69 (50.4)
No sequelae	13 (68.4)	18 (100)	18 (100)	25 (73.5)	35 (92.1)	119 (86.9)
Asymmetry of paralysis	5 (26.3)	8 (44.4)	14 (50.0)	28 (82.4)	27 (71.1)	82 (59.9)

Values are presented as number (%).

**Table 2.** Mean annual incidence rate of non-polio AFP per 100,000 under 15-year-old children by age and gender using Poisson regression analysis

Variable	n (%)	Population	AFP rate	RR	95% CI	p-value
Age (yr)						
<5	72 (52.6)	1,989,008	3.62	1.00	-	-
5-9	38 (27.7)	1,873,785	2.03	0.56	0.39, 0.83	0.002
10-14	27 (19.7)	2,155,154	1.25	0.35	0.22, 0.54	0.001
Gender						
Men	73 (53.3)	3,061,275	2.37	1.00	-	-
Women	64 (46.7)	2,926,685	2.18	0.92	0.65, 1.29	0.34

AFP, acute flaccid paralysis; RR, relative risk; CI, confidence interval.
